# Oral Nicotinamide for the Secondary Prevention of Cutaneous Squamous Cell Carcinoma and Other Keratinocyte Carcinomas: A Systematic Review

**DOI:** 10.1155/jskc/8756577

**Published:** 2026-10-05

**Authors:** Andrea Lizeth Guerra Jiménez, Juan Carlos Ortegón Garciaherreros, Lilia Patricia Suárez Escobar, Laura Valentina Vargas Monroy, María Camila Madariaga Estupiñán

**Affiliations:** ^1^ Faculty of Health Sciences Fundación Universitaria San Martín Bogotá D.C. Colombia; ^2^ School of Medicine and Health Sciences Universidad del Rosario Bogotá D.C. Colombia urosario.edu.co; ^3^ Faculty of Medicine Fundación Universitaria de Ciencias de la Salud Bogotá D.C. Colombia fucsalud.edu.co; ^4^ Faculty of Medicine Universidad Nacional de Colombia Bogotá D.C. Colombia unal.edu.co; ^5^ Department of Public Health Universidad del Norte Barranquilla Atlántico Colombia uninorte.edu.co

**Keywords:** cutaneous squamous cell carcinoma, keratinocyte carcinoma, niacinamide, nicotinamide, nonmelanoma skin cancer, secondary prevention, solid organ transplantation, systematic review

## Abstract

**Background:**

Oral nicotinamide has been proposed as a chemoprevention strategy to reduce new keratinocyte carcinomas in people with a history of skin cancer, but its effect may differ between immunocompetent patients and transplant recipients.

**Objective:**

To evaluate the effectiveness and safety of oral nicotinamide for the secondary prevention of cutaneous squamous cell carcinoma and other keratinocyte carcinoma–related outcomes in adults with a history of skin cancer.

**Methods:**

We conducted a systematic review following PRISMA 2020. We searched PubMed/MEDLINE, Embase, and LILACS through the Virtual Health Library from inception to May 30, 2026. We included comparative studies (randomized clinical trials and controlled nonrandomized studies) evaluating oral nicotinamide versus placebo, no treatment, usual care, or another preventive strategy. Risk of bias was assessed with RoB 2 for randomized trials and ROBINS‐I for nonrandomized studies, and the certainty of evidence was assessed with GRADE. We performed a narrative synthesis and an exploratory random‐effects meta‐analysis of randomized trials when clinically appropriate; effect measures were rate ratios for randomized trials and hazard ratios for the observational cohort. Randomized and nonrandomized designs were not statistically pooled together.

**Results:**

We identified 1076 records; after removing 151 duplicates, 925 records were screened, 17 full texts were assessed, and 5 studies were included: 4 randomized clinical trials and 1 large retrospective cohort. In high‐risk immunocompetent adults, the benefit signal was driven by a single pivotal randomized trial (ONTRAC; *n* = 386), in which nicotinamide reduced new nonmelanoma skin cancers (rate ratio, 0.77; 95% CI, 0.62–0.96). In transplant recipients, the main exploratory meta‐analysis of two randomized trials (*n* = 180) showed no significant reduction in total keratinocyte carcinoma/nonmelanoma skin cancer (pooled rate ratio, 0.97; 95% CI, 0.77–1.22; *I* ^2^ = 0.0%), cSCC/SCC (0.90; 95% CI, 0.65–1.25; 2 trials), or basal cell carcinoma (0.80; 95% CI, 0.20–3.18; 2 trials). A large retrospective cohort (Breglio et al.; 33,822 patients) reported an overall 14% reduction in skin cancer (hazard ratio, 0.86; 95% CI, 0.83–0.89) that was greatest for cSCC and greatest when nicotinamide was started after the first skin cancer, but there was no overall benefit in transplant recipients (hazard ratio, 1.02; 95% CI, 0.84–1.25). Certainty was low or very low for the transplant–recipient outcomes.

**Conclusions:**

In high‐risk immunocompetent adults, oral nicotinamide may reduce new nonmelanoma skin cancers during active treatment, but this conclusion rests predominantly on one large randomized trial and should be regarded as provisional. In transplant recipients, the randomized evidence does not demonstrate a significant benefit, a finding concordant with the overall null result in the large observational cohort. An observational signal favoring early initiation is hypothesis‐generating and requires prospective confirmation.

**Limitations:**

Evidence was limited by the very small number of randomized trials (only two contributed to the main meta‐analysis), imprecision, follow‐up of 12 months or less in all randomized trials, the observational nature of the largest study, and low or very low certainty for the transplant–recipient outcomes.

## 1. Introduction

Keratinocyte carcinomas, comprising mainly basal cell carcinoma and cutaneous squamous cell carcinoma, account for most skin cancers and constitute a relevant clinical problem because of their high frequency, the possibility of multiple tumors, recurrence, and the need for prolonged dermatologic surveillance. In the most recent global estimates, GLOBOCAN 2022 reported approximately 20 million new cancer cases and 9.7 million cancer deaths worldwide. Within this burden, nonmelanoma skin cancer accounted for approximately 1,234,600 new cases and 69,500 deaths; these figures should be interpreted with caution because incidence excludes basal cell carcinoma, whereas mortality includes all types of nonmelanoma skin cancer [1].

Cutaneous squamous cell carcinoma is of particular importance among keratinocyte carcinomas because of its capacity for local invasion, recurrence, metastasis, and disease‐specific mortality in high‐risk subgroups. Management guidelines for cutaneous squamous cell carcinoma emphasize that, although most cases can be treated locally, advanced disease can cause significant morbidity, impair quality of life, and lead to death [2].

Factors associated with worse prognosis include immunosuppression, head and neck location, recurrent tumors, larger size or greater tumor depth, poor histologic differentiation, perineural invasion, and deep invasion [2, 3]. In solid organ transplant recipients, chronic immunosuppression substantially modifies the natural history of skin cancer. These patients have a 50‐ to 100‐fold higher risk of cutaneous squamous cell carcinoma than the immunocompetent population, frequently develop multiple tumors, and may accumulate a higher lifetime risk of metastasis [3].

Despite the clinical importance of secondary prevention, uncertainty persists regarding the true magnitude of benefit of oral nicotinamide for preventing new cutaneous squamous cell carcinomas and other keratinocyte carcinomas in adults with a history of skin cancer. Nicotinamide, the amide form of vitamin B3 and a precursor of nicotinamide adenine dinucleotide (NAD+), has been proposed as a chemopreventive intervention because of its potential to enhance repair of ultraviolet radiation–induced DNA damage, preserve cellular energy metabolism, and reduce ultraviolet radiation–induced cutaneous immunosuppression [4, 5].

The available clinical evidence has shown heterogeneous results between immunocompetent populations and solid organ transplant recipients. This variability suggests that the magnitude of the preventive effect may depend on immune status, baseline risk, the type of outcome assessed, and the duration of follow‐up. The debate has intensified following the neutral result of the ONTRANS trial in transplant recipients and the publication of a large real‐world cohort, and recent critical appraisals have argued that the evidence remains inconclusive [6]. It is therefore necessary to critically synthesize the available evidence, separating clinically relevant populations and outcomes and distinguishing randomized from observational designs.

The objective of this systematic review was to evaluate the effectiveness and safety of oral nicotinamide for the secondary prevention of cutaneous squamous cell carcinoma and other keratinocyte carcinoma–related outcomes in adults with a history of skin cancer.

## 2. Methods

### 2.1. Design and Reporting

We conducted a systematic review according to a predefined protocol and following the PRISMA 2020 statement for systematic reviews [7]. The research question was structured using the PICOS framework to assess whether oral nicotinamide reduces the occurrence of new cutaneous squamous cell carcinoma events and other keratinocyte carcinoma‐related outcomes in adults with a history of skin cancer. The review protocol was registered in the International Prospective Register of Systematic Reviews (PROSPERO; Registration Number CRD420261407650, Version 1.0). No important deviations from the registered protocol were made, except that the planned prediction interval was not calculated because only two studies contributed to each meta‐analysis; this deviation is reported in the statistical methods.

### 2.2. Eligibility Criteria

Studies conducted in adults aged 18 years or older with a history of cutaneous squamous cell carcinoma, basal cell carcinoma, keratinocyte carcinoma, or nonmelanoma skin cancer were eligible. Both immunocompetent and immunosuppressed populations were included, including solid organ transplant recipients. Studies that included participants younger than 18 years were excluded when separate data for adults were not available.

The intervention of interest was oral nicotinamide, also called niacinamide, administered at any dose, frequency, or duration. Eligible comparators included placebo, absence of nicotinamide, usual care, photoprotection, field therapies, or other preventive strategies when present as a comparator group.

The primary outcome was the incidence of new or recurrent cutaneous squamous cell carcinoma. Related outcomes were also considered, including total keratinocyte carcinoma, nonmelanoma skin cancer, basal cell carcinoma, actinic keratoses, time to new skin cancer, tumor count, adverse events, treatment discontinuation, and tolerability.

We included randomized clinical trials, nonrandomized clinical trials, prospective cohorts, retrospective cohorts, and comparative real‐world studies. For nonrandomized studies, eligibility required a concurrent external comparator group; uncontrolled before–after studies were excluded. We excluded reviews, editorials, letters without original data, case reports, case series without a comparator, cross‐sectional studies without follow‐up, preclinical studies, registries without published results, studies evaluating only topical nicotinamide, and studies on other vitamin B3 derivatives without separate data for oral nicotinamide.

### 2.3. Information Sources and Search Strategy

We performed a systematic search in PubMed/MEDLINE, Embase, and LILACS through the Virtual Health Library (VHL), from database inception to May 30, 2026. The strategy combined terms related to nicotinamide or niacinamide, cutaneous squamous cell carcinoma, basal cell carcinoma, keratinocyte carcinoma, nonmelanoma skin cancer, actinic keratosis, chemoprevention, and secondary prevention. No language restrictions or study design filters were applied. The complete search strategies, platforms, search dates, limits, and numbers of records retrieved are presented in the Supporting Methods.

The identified records were exported, organized by database, and deduplicated before title and abstract screening. The complete selection process was summarized using a PRISMA 2020 flow diagram [7].

### 2.4. Study Selection

Two reviewers independently assessed titles and abstracts against the eligibility criteria. Records considered potentially eligible proceeded to full‐text review by two reviewers. Discrepancies and uncertain decisions were resolved by consensus or adjudication by a third reviewer. The reports were excluded at the full‐text stage, and the reasons for exclusion are presented in Supporting Table 1.

After removing duplicates, 925 records were screened by title and abstract. Seventeen full texts were sought and assessed for eligibility, all of which were retrieved. After eligibility review, 12 reports were excluded and 5 studies (4 randomized clinical trials and 1 retrospective cohort) were included in the final review.

### 2.5. Data Extraction

Data were extracted using a standardized matrix and independently checked by a second reviewer; discrepancies were resolved by consensus. We collected information on author, year of publication, country, study design, clinical setting, population, immune status, inclusion criteria, sample size, intervention, nicotinamide dose and duration, comparator, follow‐up duration, outcomes assessed, effect estimates, confidence intervals, adverse events, losses to follow‐up, and main methodological limitations.

For tumor count outcomes in randomized trials, rate ratios were used as the common effect measure. When a study reported a relative rate difference (RRD), it was transformed to a rate ratio as RR = 1 − RRD; confidence limits were transformed as 1 minus the corresponding opposite RRD limit. Estimates and 95% confidence intervals were converted to the logarithmic scale, and standard errors were calculated as [ln(upper limit) − ln(lower limit)]/(2 × 1.96). For the retrospective cohort, adjusted hazard ratios and 95% confidence intervals were extracted as reported and were kept separate from the rate ratio synthesis. The dataset and derivations used for the meta‐analyses are presented in Supporting Table 4.

### 2.6. Risk‐of‐Bias Assessment

The risk of bias of the included randomized clinical trials was assessed independently by two reviewers using the Cochrane Risk of Bias 2 (RoB 2) tool for the effect of assignment to intervention [8]. We evaluated the domains of bias arising from the randomization process, deviations from intended interventions, missing outcome data, outcome measurement, and selection of the reported result. Each result was classified as low risk of bias, some concerns, or high risk of bias, and the overall judgment followed the RoB 2 algorithm [8]. The included nonrandomized (retrospective cohort) study was assessed with the ROBINS‐I tool across its seven domains (confounding, selection of participants, classification of interventions, deviations from intended interventions, missing data, measurement of outcomes, and selection of the reported result), with an overall judgment of low, moderate, serious, or critical risk of bias [9]. Disagreements were resolved by consensus or third‐reviewer adjudication. Outcome‐specific domain judgments are presented in Supporting Tables 2 and 2B.

### 2.7. Data Synthesis and Statistical Analysis

A structured narrative synthesis of all included studies was performed, following the Synthesis Without Meta‐Analysis (SWiM) guideline when quantitative combination was not appropriate [10]. Results were grouped according to participants’ immune status, study design, type of prior skin cancer, nicotinamide dose, comparator, outcome type, and follow‐up duration. Within each group, the direction and magnitude of effect, the risk of bias, and the consistency of findings were described. The grouping criteria and the feasibility of each planned analysis are detailed in Supporting Table 3.

Meta‐analysis was considered when at least two studies were clinically and methodologically comparable with respect to design, immune status, prior skin cancer history, nicotinamide regimen, comparator, outcome definition, and follow‐up duration. Randomized and nonrandomized designs were not statistically combined; the large retrospective cohort was therefore synthesized narratively and was not pooled with the randomized trials, in line with GRADE guidance to consider study designs separately. Because of the clinical heterogeneity between immunocompetent patients and solid organ transplant recipients, these populations were not combined in the main analysis. The main exploratory quantitative analysis was restricted to randomized trials in transplant recipients for the outcome of total keratinocyte carcinoma or nonmelanoma skin cancer.

Rate ratios were pooled on the logarithmic scale using a DerSimonian–Laird random‐effects model [11]. A random‐effects model rather than a fixed‐effect model was prespecified because the a priori clinical expectation was that true effects could vary across trials differing in immune status, baseline risk, and follow‐up duration; the DerSimonian–Laird estimator was chosen as the conventional, widely reported method that permits comparison with previous reviews. Because meta‐analyses restricted to two studies are known to yield unstable estimates of between‐study variance, we additionally re‐estimated each pooled effect with restricted maximum likelihood (REML) for the heterogeneity variance and with the Hartung–Knapp–Sidik–Jonkman (HKSJ) adjustment [12] as post hoc robustness analyses added during revision; these analyses were not prespecified in the protocol. With only two studies, classical HKSJ intervals can become either markedly wider or, when residual heterogeneity is very small, spuriously narrower than conventional intervals; they were therefore used only to assess the robustness of the qualitative interpretation. The results are reported in Supporting Table 4C and summarized in the Results. Results were back‐transformed and presented as pooled rate ratios with 95% confidence intervals. Statistical heterogeneity was assessed using *I* ^2^ and *τ* ^2^ [13]. Prediction intervals were not calculated because of the small number of studies per meta‐analysis. Because fewer than 10 studies contributed to each pooled outcome, funnel plots and formal tests for publication bias were not performed; this risk was discussed narratively.

The cutaneous squamous cell carcinoma/SCC and basal cell carcinoma outcomes in transplant recipients were analyzed as exploratory. Sensitivity analyses combining all populations for total KC/NMSC and cSCC/SCC were also performed; these were not considered main analyses because of the clinical heterogeneity between immunocompetent patients and transplant recipients. The figures for these exploratory and sensitivity analyses are presented as Supporting Figures 1 to 4. The full set of planned subgroup and sensitivity analyses, together with how each was handled, is summarized in Supporting Table 6.

### 2.8. Certainty of Evidence

The certainty of evidence was assessed using the GRADE approach, considering risk of bias, inconsistency, indirectness, imprecision, and publication bias [14]. The prioritized outcomes were cutaneous squamous cell carcinoma/SCC, total keratinocyte carcinoma or nonmelanoma skin cancer, basal cell carcinoma, actinic keratoses, adverse events, and discontinuation due to adverse events. The detailed GRADE profile is presented in Supporting Table 5.

## 3. Results

### 3.1. Study Selection

The search identified 1076 records across the databases. After removing 151 duplicates, 925 records were screened by title and abstract. Of these, 908 were excluded for not meeting the eligibility criteria. Seventeen full texts were sought and assessed for eligibility. After eligibility review, 12 reports were excluded and 5 studies (4 randomized clinical trials and 1 retrospective cohort) were included in the final systematic review. The complete selection process is summarized in Figure 1.

**FIGURE 1:**
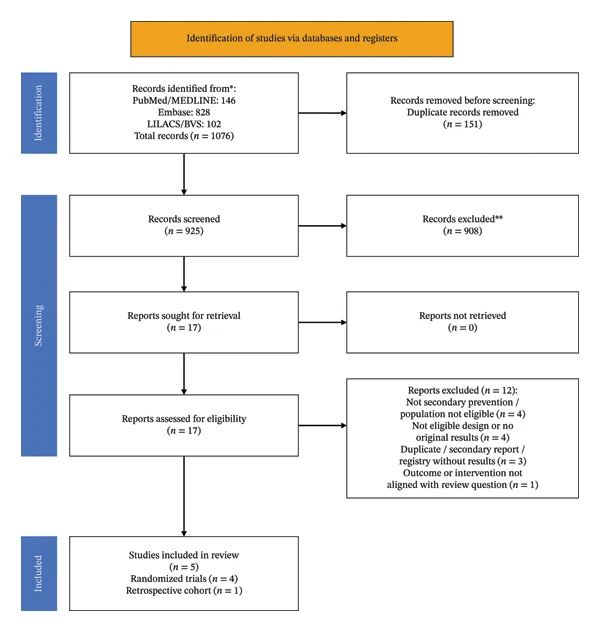
PRISMA 2020 flow diagram of study selection. The diagram shows the identification, screening, eligibility assessment, and inclusion of studies evaluating oral nicotinamide for the secondary prevention of keratinocyte carcinoma/cutaneous squamous cell carcinoma. Of 17 reports assessed for eligibility, 12 were excluded and 5 studies were included (4 randomized clinical trials and 1 retrospective cohort) [7].

Of the five included studies, the ONTRAC randomized trial provided the immunocompetent efficacy data, two randomized trials in transplant recipients contributed to the main exploratory meta‐analysis, one randomized pilot trial was retained for secondary narrative synthesis because it mainly evaluated actinic keratoses and tolerability, and one large retrospective cohort provided real‐world observational evidence in both the general (predominantly immunocompetent) population and transplant recipients.

### 3.2. Characteristics of Included Studies

The main characteristics of the included studies are presented in Table 1. Four of the five included studies were randomized, double‐blind, placebo‐controlled clinical trials, and one was a large retrospective cohort study. One trial included high‐risk immunocompetent adults with a history of nonmelanoma skin cancer, two trials evaluated transplant recipients with keratinocyte carcinoma or nonmelanoma skin cancer outcomes, one pilot trial in renal transplant recipients primarily evaluated actinic keratoses and tolerability, and one retrospective cohort evaluated a large mixed population with a history of skin cancer [4, 5, 15–17].

**TABLE 1:** Characteristics of the included studies.

Study	Design	Population	*N*	Intervention	Comparator	Follow‐up	Main outcomes
Chen et al., 2015/ONTRAC [4]	Randomized, double‐blind, placebo‐controlled trial	High‐risk immunocompetent adults with a history of ≥ 2 NMSC in the previous 5 years	386	Oral nicotinamide 500 mg twice daily	Placebo	12 months	NMSC, SCC/cSCC, BCC, actinic keratoses, safety
Allen et al., 2023/ONTRANS [5]	Randomized, double‐blind, placebo‐controlled trial	Solid organ transplant recipients with a history of ≥ 2 KC in the previous 5 years	158	Oral nicotinamide 500 mg twice daily	Placebo	12 months	KC, SCC/cSCC, BCC, actinic keratoses, safety
Chen et al., 2016 [15]	Phase II randomized, double‐blind, placebo‐controlled trial	Renal transplant recipients with a history of nonmelanoma skin cancer	22	Oral nicotinamide 500 mg twice daily	Placebo	6 months	NMSC, SCC/cSCC, BCC, safety
Zhang et al., 2023 [16]	Randomized, double‐blind, placebo‐controlled pilot trial	Renal transplant recipients with a history of KC and actinic keratoses	21	Oral nicotinamide; dose modified during the study for tolerability	Placebo	12 months	Actinic keratoses, tolerability, adverse events
Breglio et al., 2025 [17]	Retrospective cohort (propensity score matched); STROBE‐reported	Adults (U.S. Veterans Affairs) with ≥ 1 prior skin cancer; predominantly immunocompetent, 3.9% transplant recipients	33,822 (12,287 exposed; 21,535 matched unexposed)	Oral nicotinamide 500 mg twice daily for ≥ 30 days (EHR‐documented)	Propensity‐matched unexposed patients	Time to next skin cancer (up to years)	Skin cancer overall, cSCC, BCC; SOTR subgroup; dose–response

Abbreviations: BCC, basal cell carcinoma; cSCC, cutaneous squamous cell carcinoma; EHR, electronic health record; KC, keratinocyte carcinoma; NMSC, nonmelanoma skin cancer; SCC, squamous cell carcinoma; SOTR, solid organ transplant recipient; STROBE, Strengthening the Reporting of Observational Studies in Epidemiology.

In the immunocompetent population, the ONTRAC trial evaluated oral nicotinamide 500 mg twice daily versus placebo for 12 months in adults with a history of at least two nonmelanoma skin cancers in the previous 5 years [4]. In transplant recipients, ONTRANS evaluated the same nicotinamide dose versus placebo for 12 months in solid organ recipients with a history of at least two keratinocyte carcinomas in the previous 5 years [5]. A Phase II trial in renal transplant recipients evaluated nicotinamide 500 mg twice daily versus placebo for 6 months [15]. A pilot trial in renal transplant recipients evaluated oral nicotinamide for the prevention of actinic keratoses and was therefore synthesized narratively and not included in the main meta‐analysis [16]. Finally, the retrospective cohort by Breglio et al. used electronic health record data from the U.S. Veterans Affairs system (1999–2024) and included 33,822 patients with at least one prior skin cancer; 12,287 patients exposed to oral nicotinamide 500 mg twice daily for at least 30 days were propensity score matched to 21,535 unexposed patients on the number and timing of prior skin cancers and other covariates, and the outcome was time to the next skin cancer, analyzed with stratified Cox models [17].

### 3.3. Risk of Bias

The risk‐of‐bias assessment of the randomized trials is summarized in Figure 2. Two randomized trials were classified as low risk of bias, one as some concerns and one as high risk of bias. The ONTRAC trial and the Phase II trial in renal transplant recipients were classified as low risk of bias [4, 15]. The ONTRANS trial was classified as some concerns, mainly because of aspects related to the modified intention‐to‐treat analysis and incomplete follow‐up [5]. The pilot trial by Zhang et al. was classified as high risk of bias because of relevant dose modifications, differential losses, and a very small sample size [16]. The visualization was generated using robvis [18].

**FIGURE 2:**
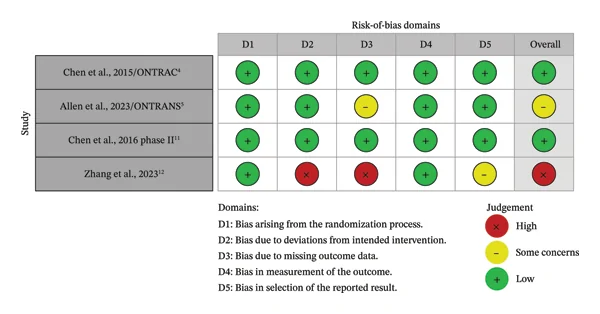
Risk‐of‐bias assessment of the included randomized clinical trials using the Cochrane Risk of Bias 2 tool. Risk of bias was assessed across five domains: D1, bias arising from the randomization process; D2, bias due to deviations from intended interventions; D3, bias due to missing outcome data; D4, bias in outcome measurement; and D5, bias in selection of the reported result. Green circles indicate low risk of bias, yellow indicates some concerns, and red indicates high risk of bias. The nonrandomized study (Breglio et al.) was assessed separately with ROBINS‐I and is reported in Supporting Table 2B. The visualization was generated using robvis [8, 9, 18].

The retrospective cohort by Breglio et al. was assessed with ROBINS‐I and judged to be at serious overall risk of bias, driven principally by the domains of confounding (residual and unmeasured confounding despite propensity score matching, as acknowledged by the authors; matching balance was not achieved for patients treated after nine or more prior skin cancers, and nicotinamide‐exposed patients were more likely to have higher‐risk features such as chronic lymphocytic leukemia and acitretin exposure, a confounding by indication that would bias toward the null), and classification of interventions (ascertainment of nicotinamide exposure from prescription records, with possible over‐the‐counter or non–Veterans Affairs use in the comparator group, which the authors note may have underestimated the true effect). Additional considerations included the exclusion of skin cancers treated within 90 days of baseline (introducing immortal‐time bias by design) and the use of administrative ICD/CPT codes rather than histology to classify tumor type. Several of these biases were expected by the authors to bias the estimate toward the null, so the observed protective associations are unlikely to be artifacts of these limitations. Domain‐level ROBINS‐I judgments are presented in Supporting Table 2B.

### 3.4. Results by Study

The main results of the included studies are presented in Table 2. In the high‐risk immunocompetent population, oral nicotinamide reduced the rate of new nonmelanoma skin cancers during the intervention period [4]. In contrast, in solid organ transplant recipients, ONTRANS showed no significant reduction in new keratinocyte carcinomas, cSCC/SCC, BCC, or actinic keratoses with nicotinamide versus placebo [5]. The Phase II trial in renal transplant recipients suggested a nonsignificant reduction in total nonmelanoma skin cancer but had a small sample size and imprecise estimates [15]. The retrospective cohort by Breglio et al. reported an overall 14% reduction in new skin cancers (adjusted hazard ratio, 0.86; 95% CI, 0.82–0.89), no overall reduction for basal cell carcinoma (1.00; 95% CI, 0.96–1.04), and a reduction for cutaneous squamous cell carcinoma (0.79; 95% CI, 0.76–0.82); the protective association was largest when nicotinamide was initiated after the first skin cancer (a 54% reduction in overall skin cancer; after‐first‐cancer hazard ratios of 0.44 for any skin cancer and 0.47 for cSCC) and attenuated progressively with each subsequent skin cancer, disappearing beyond the sixth. Among transplant recipients, there was no significant overall association (1.02; 95% CI, 0.84–1.25), although a significant reduction in cSCC was observed in the subgroup with two or fewer prior skin cancers (0.47; 95% CI, 0.23–0.97) [17].

**TABLE 2:** Main results by study.

Study	Population	Outcome	Main result	Interpretation
Chen 2015/ONTRAC [4]	High‐risk immunocompetent	Total NMSC	Rate ratio, 0.77; 95% CI, 0.62–0.96	Significant reduction during the treatment period
Chen 2015/ONTRAC [4]	High‐risk immunocompetent	SCC/cSCC	Rate ratio, 0.70; 95% CI, 0.49–1.00	Borderline reduction favoring nicotinamide
Chen 2015/ONTRAC [4]	High‐risk immunocompetent	BCC	Reduction not statistically significant	Less clear benefit for BCC
Allen 2023/ONTRANS [5]	Solid organ transplant recipients	Total KC	207 vs 210 lesions; rate ratio, 1.00; 95% CI, 0.80–1.30	No significant reduction
Allen 2023/ONTRANS [5]	Solid organ transplant recipients	SCC/cSCC	138 vs 151 lesions; rate ratio, 0.90; 95% CI, 0.60–1.20	No significant reduction
Allen 2023/ONTRANS [5]	Solid organ transplant recipients	BCC	69 vs 59 lesions; rate ratio, 1.40; 95% CI, 0.80–2.30	No benefit demonstrated
Chen 2016 [15]	Renal transplant recipients	Total NMSC	30 vs 45 lesions; estimate, 0.65; 95% CI, 0.30–1.60	Nonsignificant reduction; small study
Chen 2016 [15]	Renal transplant recipients	SCC/cSCC	23 vs 24 lesions; approximate estimate, 0.93; 95% CI, 0.35–2.51	No significant reduction; imprecise
Chen 2016 [15]	Renal transplant recipients	BCC	7 vs 21 lesions; estimate, 0.33; 95% CI, 0.08–1.45	Nonsignificant reduction; very imprecise
Zhang 2023 [16]	Renal transplant recipients	Actinic keratoses	Mean at 12 months: 30.8 (nicotinamide) vs 26.6 (placebo); *p* = 0.38	No significant difference
Zhang 2023 [16]	Renal transplant recipients	Tolerability	Differential losses (∼40% vs 0%) and dose reduction due to gastrointestinal events	Evidence limited by high risk of bias
Breglio 2025 [17]	Overall cohort (predominantly immunocompetent)	Any skin cancer	Adjusted HR 0.86 (95% CI, 0.83–0.89) (14% reduction)	Significant overall reduction; observational
Breglio 2025 [17]	Overall cohort	cSCC	Adjusted HR, 0.79; 95% CI, 0.76–0.82	Significant reduction, largest for cSCC
Breglio 2025 [17]	Overall cohort	BCC	Adjusted HR, 1.00; 95% CI, 0.96–1.04	No overall reduction for BCC
Breglio 2025 [17]	Overall cohort (early initiation)	Any skin cancer/cSCC	54% reduction in overall skin cancer when started after the first skin cancer (any‐cancer HR, 0.44; cSCC, HR 0.47); attenuates with each subsequent cancer	Timing‐dependent effect; hypothesis‐generating
Breglio 2025 [17]	Solid organ transplant recipients	Any skin cancer	Adjusted HR, 1.02; 95% CI, 0.84–1.25	No significant overall reduction
Breglio 2025 [17]	SOTR with ≤ 2 prior skin cancers	cSCC	Adjusted HR, 0.47; 95% CI, 0.23–0.97 (*n* = 412)	Significant reduction in early treated subgroup

*Note:* For randomized trials, effect measures are rate ratios; for the retrospective cohort, adjusted hazard ratios. For Breglio et al. [17], the overall hazard‐ratio estimates were extracted from Figure 4 of the source article (BCC, 1.00; 95% CI, 0.96–1.04; cSCC, 0.79; 95% CI, 0.76–0.82). The narrative Results paragraph of the source article reports slightly different rounded values; the corrected figure was used consistently for extraction.

Abbreviations: BCC, basal cell carcinoma; cSCC, cutaneous squamous cell carcinoma; CI, confidence interval; HR, hazard ratio; KC, keratinocyte carcinoma; NMSC, nonmelanoma skin cancer; SCC, squamous cell carcinoma; SOTR, solid organ transplant recipient.

### 3.5. Quantitative Synthesis

The quantitative synthesis is summarized in Table 3. The main exploratory meta‐analysis restricted to randomized trials in transplant recipients included two clinically comparable trials (ONTRANS and the Phase II trial; 180 participants). For total keratinocyte carcinoma or nonmelanoma skin cancer, the pooled effect was a rate ratio of 0.97 (95% CI, 0.77–1.22), with no observed statistical heterogeneity (*I* ^2^ = 0.0%; *τ* ^2^ = 0.000). This result showed no significant reduction with oral nicotinamide versus placebo in transplant recipients. The analysis is shown in Figure 3.

**TABLE 3:** Quantitative synthesis of results (randomized trials).

Analysis	Included studies	Population	Outcome	Pooled effect (DerSimonian–Laird)	Heterogeneity	Interpretation
Main exploratory meta‐analysis	Allen 2023; Chen 2016 [5, 11]	Transplant recipients	Total KC/NMSC	Rate ratio, 0.97; 95% CI, 0.77–1.22	*I* ^2^ = 0.0%; *τ* ^2^ = 0.000	No significant reduction with nicotinamide
Exploratory analysis	Allen 2023; Chen 2016 [5, 11]	Transplant recipients	SCC/cSCC	Rate ratio, 0.90; 95% CI, 0.65–1.25	*I* ^2^ = 0.0%; *τ* ^2^ = 0.000	No significant reduction; cautious interpretation
Exploratory analysis	Allen 2023; Chen 2016 [5, 11]	Transplant recipients	BCC	Rate ratio, 0.80; 95% CI, 0.20–3.18	*I* ^2^ = 70.4%; *τ* ^2^ = 0.735	Very imprecise and heterogeneous; not a main outcome
Sensitivity analysis	Chen 2015; Allen 2023; Chen 2016 [4, 5, 11]	All populations	Total KC/NMSC	Rate ratio, 0.86; 95% CI, 0.69–1.06	*I* ^2^ = 30.9%; *τ* ^2^ = 0.011	Nonsignificant trend; not a main analysis (clinical heterogeneity)
Sensitivity analysis	Chen 2015; Allen 2023; Chen 2016 [4, 5, 11]	All populations	SCC/cSCC	Rate ratio, 0.80; 95% CI, 0.63–1.02	*I* ^2^ = 0.0%; *τ* ^2^ = 0.000	Nonsignificant trend; not a main analysis (mixed populations)

*Note:* Values below 1 favor nicotinamide. All pooled estimates use rate ratios and the DerSimonian–Laird random‐effects model; robustness to the REML and Hartung–Knapp–Sidik–Jonkman estimators is reported in Supporting Table 4C. The large retrospective cohort [17] was not pooled with the randomized trials because of design and effect measure differences and is synthesized narratively.

Abbreviations: BCC, basal cell carcinoma; cSCC, cutaneous squamous cell carcinoma; CI, confidence interval; KC, keratinocyte carcinoma; NMSC, nonmelanoma skin cancer; SCC, squamous cell carcinoma.

**FIGURE 3:**
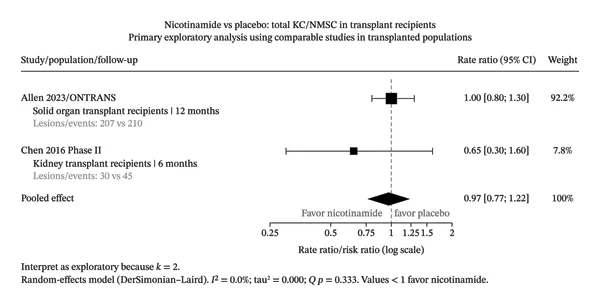
Forest plot of oral nicotinamide versus placebo for total keratinocyte carcinoma/nonmelanoma skin cancer in transplant recipients. Random‐effects (DerSimonian–Laird) meta‐analysis of two comparable randomized clinical trials in transplant recipients (ONTRANS and the Phase II renal transplant trial; 180 participants in total). Estimates are rate ratios with 95% confidence intervals; values below 1 favor nicotinamide. Lesion counts (nicotinamide vs placebo) were 207 vs 210 in ONTRANS and 30 vs 45 in the Phase II trial. Study weights were 92.2% (ONTRANS) and 7.8% (Phase II trial). The pooled effect was a rate ratio of 0.97 (95% CI, 0.77–1.22), with no observed statistical heterogeneity (*I* ^2^ = 0.0%; *τ* ^2^ = 0.000). This analysis should be interpreted as exploratory because it included only two studies; sensitivity analyses using REML and Hartung–Knapp–Sidik–Jonkman estimators are reported in Supporting Table 4C [5, 11–13, 15].

Because this analysis was based on only two studies, we examined the robustness of the pooled estimate to the choice of statistical method. Given that the estimated between‐study variance was zero (*I* ^2^ = 0.0%; *τ* ^2^ = 0.000), the REML estimator produced an identical pooled rate ratio of 0.97 (95% CI, 0.77–1.22). The HKSJ adjustment left the point estimate unchanged (0.97) but, as expected when only two studies are available and the reference distribution has a single degree of freedom, produced a substantially wider and uninformative interval (95% CI, 0.22–4.19). Thus, the qualitative conclusion of no demonstrated benefit was consistent across estimators, but the width and stability of the interval depended heavily on the two‐study evidence base, underscoring the fragility of the pooled estimate. Full estimator comparisons for all pooled outcomes are provided in Supporting Table 4C.

The exploratory analyses of cSCC/SCC and BCC in transplant recipients are presented in the Supporting Information. For cSCC/SCC, the pooled effect was 0.90 (95% CI, 0.65–1.25; *I* ^2^ = 0.0%; 2 trials), whereas for BCC it was 0.80 (95% CI, 0.20–3.18; *I* ^2^ = 70.4%; 2 trials). These analyses should be interpreted with caution because of the small number of studies, imprecision, and, in the case of BCC, heterogeneity.

### 3.6. Observational Evidence

The large retrospective cohort by Breglio et al. was not pooled with the randomized trials because of the difference in study design and effect measure (adjusted hazard ratios rather than tumor count rate ratios). Considered narratively, its findings are directionally concordant with the randomized evidence: an overall protective association in a predominantly immunocompetent, high‐risk population that is strongest for cSCC and when treatment is started early, alongside no overall benefit in transplant recipients. The much larger sample size increases precision relative to the trials but does not overcome the intrinsic limitations of an observational design at serious risk of bias, so it strengthens rather than establishes the immunocompetent benefit signal and reinforces the neutral transplant–recipient conclusion [17].

### 3.7. Sensitivity Analyses

The sensitivity analyses combining the immunocompetent population and transplant recipients (randomized trials only) are presented in the Supporting Information. For total keratinocyte carcinoma or nonmelanoma skin cancer across all populations, the pooled effect was 0.86 (95% CI, 0.69–1.06; *I* ^2^ = 30.9%; three trials). For cSCC/SCC across all populations, the pooled effect was 0.80 (95% CI, 0.63–1.02; *I* ^2^ = 0.0%; three trials). These analyses were not considered main analyses because of the clinical heterogeneity between populations.

### 3.8. Actinic Keratoses

Evidence on actinic keratoses was variable. In the immunocompetent population, nicotinamide showed a reduction in actinic keratoses during the intervention period [4]. In transplant recipients, the ONTRANS trial found no significant differences in actinic keratoses between nicotinamide and placebo [5].

The pilot trial in renal transplant recipients specifically evaluated actinic keratoses and tolerability. At 12 months, the mean actinic keratosis count was 30.8 in the nicotinamide group and 26.6 in the placebo group, with no statistically significant difference [16]. This study was interpreted only as secondary narrative evidence because of its small sample size, differential losses, and dose changes during follow‐up.

### 3.9. Safety and Tolerability

Overall, oral nicotinamide was well tolerated in the main randomized trials, with no clear overall safety disadvantage versus placebo [4, 5, 15]. In the ONTRAC trial (193 participants per group), no noteworthy between‐group differences were found in the number or types of adverse events during the 12‐month intervention period, and platelet counts did not differ between groups [4]. A recognized nuance concerns infections: In ONTRAC, the nicotinamide group had numerically more total infections (90 vs 73) and more grade 3–5 infections (10 vs 5) than placebo, differences that were not statistically significant, together with a significant excess of skin infections (14 vs 4; *p* < 0.05) [4]. For ONTRANS, we additionally reviewed the supporting safety data of the original ONTRANS publication [5]. The safety‐analysis population comprised 78 nicotinamide‐treated and 79 placebo‐treated participants because one participant randomized to nicotinamide received no study treatment. At least one adverse event was reported in 59/78 (75.6%) versus 54/79 (68.4%) participants, with 136 versus 150 total adverse‐event episodes. Serious adverse‐event counts were 38 versus 35 episodes. Gastrointestinal adverse events accounted for 13 versus 17 episodes and affected 15.4% versus 16.5% of participants. Infection/infestation events numbered 39 versus 36 overall and 10 versus 11 serious events, respectively; therefore, these data do not support an excess of serious infections with nicotinamide [5]. Adverse‐event‐related withdrawal or treatment discontinuation was not reported by the treatment arm in the article or its supporting information and was recorded as NR rather than inferred. The Phase II trial in renal transplant recipients reported no notable safety concerns [15]. In contrast, the pilot trial by Zhang et al. reported gastrointestinal tolerability problems that prompted dose reduction during the study and differential losses (approximately 40% in the nicotinamide group versus 0% in the placebo group) [16]. Consolidated adverse‐event data by the treatment group are presented in Supporting Table 7.

### 3.10. Certainty of Evidence

The certainty of evidence is summarized in Table 4. For total keratinocyte carcinoma or nonmelanoma skin cancer in transplant recipients, certainty was low, mainly because of risk of bias in the dominant study and imprecision. For cSCC/SCC in transplant recipients, certainty was also low because of imprecision and the use of an approximate estimate in one of the studies. For BCC in transplant recipients, certainty was very low because of very serious imprecision and inconsistency. The evidence for actinic keratoses and safety was limited by heterogeneity between populations, the small number of studies, and, in the pilot trial, high risk of bias.

**TABLE 4:** Certainty of evidence according to GRADE.

Outcome	Population	Studies	Effect estimate	Certainty	Main reasons for downgrading	Interpretation
Total KC/NMSC	Transplant recipients	2 RCTs	0.97; 95% CI, 0.77–1.22	Low	Risk of bias; imprecision	No significant reduction demonstrated
cSCC/SCC	Transplant recipients	2 RCTs	0.90; 95% CI, 0.65–1.25	Low	Imprecision; one approximate estimate	No significant reduction demonstrated
BCC	Transplant recipients	2 RCTs	0.80; 95% CI, 0.20–3.18	Very low	Very serious imprecision; inconsistency	Highly uncertain effect
Total KC/NMSC	All populations	3 RCTs	0.86; 95% CI, 0.69–1.06	Very low	Indirectness (mixed populations); imprecision	Sensitivity analysis only
cSCC/SCC	All populations	3 RCTs	0.80; 95% CI, 0.63–1.02	Very low	Indirectness (mixed populations); imprecision	Sensitivity analysis only
Any skin cancer/cSCC	Overall cohort (observational)	1 cohort	HR, 0.86 (any); 0.79 (cSCC)	Very low	Observational design; serious risk of bias (confounding, exposure classification)	Supportive, not confirmatory
Actinic keratoses	Immunocompetent and transplant recipients	3 RCTs	Narrative synthesis	Low to very low	Risk of bias; inconsistency; imprecision	Variable findings by population
Adverse events/tolerability	Immunocompetent and transplant recipients	4 RCTs	Narrative synthesis	Low	Imprecision; heterogeneous safety reporting; AE‐related discontinuation incompletely reported	No consistent overall safety disadvantage; ONTRAC infection signal and pilot trial tolerability concerns warrant monitoring, whereas ONTRANS showed no excess of serious infection events

*Note:* GRADE starts at high certainty for randomized clinical trials (and low for observational studies) and may be downgraded for risk of bias, inconsistency, indirectness, imprecision, or publication bias.

Abbreviations: BCC, basal cell carcinoma; cSCC, cutaneous squamous cell carcinoma; CI, confidence interval; GRADE, Grading of Recommendations Assessment, Development and Evaluation; HR, hazard ratio; KC, keratinocyte carcinoma; NMSC, nonmelanoma skin cancer; RCT, randomized clinical trial; SCC, squamous cell carcinoma.

### 3.11. Overall Synthesis of Findings

Taken together, the evidence suggests that oral nicotinamide may reduce new nonmelanoma skin cancers in high‐risk immunocompetent adults during the treatment period, although this signal derives predominantly from a single pivotal randomized trial and is supported, at lower certainty, by a large observational cohort. In solid organ transplant recipients, the exploratory meta‐analysis of two randomized trials showed no significant reduction in new keratinocyte carcinomas or nonmelanoma skin cancer, a conclusion reinforced by the overall null association in the observational cohort. The certainty of these findings is limited by the small number of studies, the imprecision of the estimates, the short follow‐up, and the clinical heterogeneity between populations.

## 4. Discussion

### 4.1. Summary of Findings

In this systematic review of randomized clinical trials and one large observational cohort, oral nicotinamide showed a different effect pattern according to the immune status of the population evaluated. In high‐risk immunocompetent adults, the benefit was driven predominantly by a single pivotal trial, ONTRAC, which showed a significant reduction in new nonmelanoma skin cancers versus placebo during the active treatment period (rate ratio 0.77; 95% CI, 0.62–0.96) and a favorable, although borderline, estimate for cutaneous SCC/SCC (rate ratio 0.70; 95% CI, 0.49–1.00) [4]. Because no other randomized trial in immunocompetent adults met the eligibility criteria, this conclusion rests on one trial and must be regarded as provisional; the large retrospective cohort by Breglio et al. provides directionally consistent but lower‐certainty observational support [17]. In contrast, in solid organ transplant recipients, the exploratory meta‐analysis of two clinically comparable randomized trials showed no significant reduction in total keratinocyte carcinoma/nonmelanoma skin cancer with oral nicotinamide versus placebo [5, 15].

The pooled effect for total keratinocyte carcinoma/nonmelanoma skin cancer in transplant recipients was 0.97 (95% CI, 0.77–1.22), with low certainty of evidence, and this estimate should be read in the light of its two‐study basis. Similarly, the exploratory analyses for cSCC/SCC and basal cell carcinoma showed no statistically significant reductions. These results indicate that the possible chemopreventive effect of nicotinamide should not be assumed to be uniform across immunocompetent and immunosuppressed populations [3, 5, 6, 15, 19].

### 4.2. Clinical Heterogeneity

The included studies differed in several clinically important respects that limit their comparability and constrain evidence synthesis. Immune status was the principal source of clinical heterogeneity: ONTRAC enrolled immunocompetent adults, whereas ONTRANS, the Phase II trial, and the pilot trial enrolled transplant recipients under chronic immunosuppression, a state that alters cutaneous carcinogenesis, immune surveillance, and tumor multiplicity. The studies also differed in baseline risk (from a history of two or more skin cancers in ONTRAC and ONTRANS to a median of three, and up to more than seventy, prior cancers in the observational cohort), in follow‐up duration (6 months in the Phase II trial versus 12 months in the other trials and up to several years in the cohort), and in outcome definitions (histologically confirmed tumor counts and rate ratios in the trials versus administratively coded, procedurally treated skin cancers and hazard ratios in the cohort). The intervention regimen was consistent (500 mg twice daily) across the comparable studies, although the pilot trial required dose modification for tolerability. These differences justified separating populations and designs rather than combining them in a single estimate, and they should temper any attempt to generalize a pooled figure across the whole population of patients with prior skin cancer.

### 4.3. Agreements and Disagreements With Previous Evidence and Incremental Value

This review overlaps in topic with previous syntheses, including the systematic review and meta‐analysis by Mainville et al., the systematic review and meta‐analysis by Tosti et al., and the recent critical appraisal by Tan and Williams [6, 19, 20]. We acknowledge this overlap explicitly. Our review nevertheless offers several incremental contributions. First, it is restricted to secondary prevention in adults with a history of skin cancer, a population clinically distinct from primary prevention, whereas earlier meta‐analyses pooled more heterogeneous populations and outcomes. Second, and in contrast to reviews that combined immunocompetent patients and transplant recipients into a single estimate, we deliberately separated these populations and did not report a combined figure as a main conclusion. Third, our search extended through May 2026 and incorporated the most recent large real‐world study (Breglio et al., 2025), which was not available to the earlier meta‐analyses and which substantially increases the observational evidence base [17]. Fourth, we assessed the nonrandomized study with ROBINS‐I, kept observational and randomized evidence separate, and tested the robustness of every pooled estimate to alternative heterogeneity estimators. These features address, rather than repeat, the principal criticism raised in recent appraisals: that the evidence has often been overinterpreted by pooling dissimilar studies [6].

Our findings are consistent with the benefit signal previously observed in the high‐risk immunocompetent population. In ONTRAC, oral nicotinamide 500 mg twice daily reduced the rate of new nonmelanoma skin cancers versus placebo over 12 months (rate ratio, 0.77; 95% CI, 0.62–0.96), and the estimate for cSCC/SCC also favored nicotinamide with borderline significance (rate ratio, 0.70; 95% CI, 0.49–1.00) [4]. Mainville et al. reported an overall reduction in skin cancers with nicotinamide versus control but with substantial heterogeneity, and Tosti et al. reached broadly similar conclusions; both, however, combined populations and outcomes in ways that limit interpretation [19, 20]. In transplant recipients, ONTRANS showed no significant reduction in keratinocyte carcinomas, cSCC/SCC, basal cell carcinoma, or actinic keratoses [5], and the observational cohort likewise found no overall benefit in this population [17]. This convergence of randomized and observational evidence strengthens the conclusion that a uniform chemopreventive benefit in transplant recipients is not supported, consistent with recent critical appraisals arguing that the evidence is not yet definitive and that broad guideline adoption should be approached with caution [6].

### 4.4. Biological Plausibility

The biological plausibility of a chemopreventive effect of nicotinamide is grounded in its central role in NAD+ metabolism. As the amide form of vitamin B3 and a precursor of nicotinamide adenine dinucleotide, nicotinamide replenishes the NAD+ pool that sustains cellular ATP production; because ultraviolet radiation acutely depletes cellular energy, this replenishment supports the energy‐dependent repair of ultraviolet‐induced DNA damage and helps prevent the “energy crisis” that impairs genomic maintenance in ultraviolet‐exposed keratinocytes [21–24]. NAD+ is also a substrate for DNA repair and signaling enzymes such as poly(ADP‐ribose) polymerase 1 (PARP1) and the sirtuins, linking nicotinamide to genomic stability, and nicotinamide attenuates ultraviolet‐induced oxidative stress and reduces ultraviolet‐induced cutaneous immunosuppression, a key mechanism by which ultraviolet radiation permits tumor escape from immune surveillance [21–24]. Phase II studies also demonstrated a reduction in actinic keratoses during nicotinamide administration [25]. A recent mechanistic review synthesizes these pathways—NAD+ repletion, enhanced DNA repair, oxidative stress modulation, and reduction in ultraviolet‐related immunosuppression—as the rationale for chemoprevention and antiaging effects in the skin [24]. These mechanisms support the hypothesis of a photoprotective and chemopreventive effect; however, they do not replace direct clinical evidence, and they do not by themselves explain the divergent results between immunocompetent patients and transplant recipients, in whom pharmacologic immunosuppression may blunt any immune‐mediated component of the effect.

### 4.5. Safety and Tolerability

In the main randomized trials, oral nicotinamide was generally well tolerated, with no clear overall safety disadvantage versus placebo [4, 5, 15]. This observation is consistent with previous reviews describing nicotinamide as a generally safe intervention at the doses evaluated for photoprotection and cutaneous chemoprevention [19, 26]. Two caveats deserve emphasis in fragile, polymedicated populations. First, ONTRAC showed numerically more total and grade 3–5 infections in the nicotinamide arm and a statistically significant excess of skin infections (14 vs 4; *p* < 0.05) [4]. In ONTRANS, however, the trial’s supporting information showed 59/78 versus 54/79 participants with at least one adverse event, 38 versus 35 serious adverse‐event episodes, 13 versus 17 gastrointestinal adverse‐event episodes, and 10 versus 11 serious infection/infestation events for nicotinamide versus placebo, respectively [5]. These data do not indicate an excess of serious infections with nicotinamide. Adverse‐event‐related withdrawal or treatment discontinuation was not reported by treatment arm and therefore remains NR. Second, the pilot trial in renal transplant recipients reported gastrointestinal tolerability problems, dose modification, and substantial differential losses in the nicotinamide group [16]. These findings support continued attention to adherence, tolerability, and discontinuation in future studies, particularly in transplant recipients receiving multiple medications. Quantitative adverse‐event data are summarized in Supporting Table 7.

### 4.6. Strengths and Limitations

This review has several strengths. First, it focused specifically on secondary prevention in adults with a history of skin cancer. Second, it explicitly separated immunocompetent populations and transplant recipients and randomized from nonrandomized designs, avoiding a potentially misleading overall estimate. Third, the main exploratory meta‐analysis was restricted to clinically comparable randomized studies, and every pooled estimate was tested for robustness to alternative heterogeneity estimators. Fourth, standardized methodological tools were applied, including PRISMA 2020, RoB 2, ROBINS‐I, SWiM, and GRADE [7–10, 14, 18].

The main limitation was the small randomized evidence base. Only four randomized clinical trials met the inclusion criteria, and only two were sufficiently comparable for the main meta‐analysis in transplant recipients; a two‐study meta‐analysis provides limited reliability and precision, cannot support robust subgroup analyses or a formal assessment of publication bias, and yields between‐study variance estimates that are themselves unstable, as shown by the sensitivity analyses. The benefit in immunocompetent adults rests on a single pivotal trial. The largest study was observational and at serious risk of bias, and its hazard ratio estimates were not comparable with, or pooled with, the trial rate ratios.

All randomized trials had a follow‐up of 12 months or less, so it is not possible to establish whether the effect of nicotinamide persists after discontinuing the intervention or whether it modifies long‐term outcomes such as cumulative recurrence, metastasis, progression to advanced disease, or cutaneous squamous cell carcinoma–specific mortality; indeed, ONTRAC found no evidence of benefit after nicotinamide was discontinued [4]. Long‐term preventive effectiveness therefore cannot be inferred from the current evidence. There were also study‐specific limitations: ONTRANS had methodological concerns related to the modified intention‐to‐treat analysis and incomplete follow‐up; the Phase II trial had a small sample size; and the pilot trial by Zhang et al. had a high risk of bias [5, 15, 16].

### 4.7. Implications for Clinical Practice and Research

The results support an interpretation differentiated by immune status. In high‐risk immunocompetent adults with a history of nonmelanoma skin cancer, oral nicotinamide could be considered a complementary secondary prevention strategy during the treatment period, always integrated with photoprotection, dermatologic surveillance, and timely treatment of precursor lesions or incident tumors; this suggestion, however, is based predominantly on one randomized trial and should be presented to patients as such [4, 19, 26].

In solid organ transplant recipients, the available randomized evidence does not demonstrate a significant reduction in new keratinocyte carcinomas or nonmelanoma skin cancer, and the largest observational study found no overall benefit [5, 15, 17]. Therefore, the routine use of nicotinamide as a universal chemopreventive measure in this population is not supported with sufficient certainty. The observational finding of a benefit for cSCC when nicotinamide is initiated early (after one or two skin cancers) is biologically coherent and clinically appealing but remains hypothesis‐generating and should be tested prospectively rather than adopted as practice [17]. In these patients, the decision should be individualized, considering tumor burden, tolerability, patient preferences, the immunosuppressive regimen, and the availability of other preventive strategies. In neither group should nicotinamide be considered a substitute for photoprotection, dermatologic surveillance, early diagnosis, treatment of actinic keratoses, or comprehensive management of field cancerization [2, 3, 27].

Multicenter randomized clinical trials with an adequate sample size and follow‐up longer than 12 months are needed to define more precisely the effect of oral nicotinamide on cSCC, total keratinocyte carcinoma/nonmelanoma skin cancer, basal cell carcinoma, and actinic keratoses and to test the early initiation hypothesis directly. Future studies should stratify by immune status, transplant type, immunosuppressive regimen, number of previous skin cancers, actinic keratosis burden, and adherence and should report outcomes in a standardized manner by tumor type, person‐time rates, time to new tumor, adverse events (including infections), discontinuation, quality of life, and persistence of effect after discontinuing the intervention [6, 20, 26].

## 5. Conclusion

In high‐risk immunocompetent adults, oral nicotinamide showed benefit in reducing new nonmelanoma skin cancers during the active treatment period; however, this conclusion is based predominantly on a single pivotal randomized trial and is supported only at low certainty by observational data. In solid organ transplant recipients, the exploratory meta‐analysis of two randomized trials showed no significant reduction in total keratinocyte carcinoma/nonmelanoma skin cancer, cSCC/SCC, or basal cell carcinoma, a conclusion reinforced by the overall null association in a large observational cohort. The certainty of evidence was low or very low for the main outcomes in transplant recipients, mainly because of imprecision, the small number of studies, and methodological limitations, and no long‐term effectiveness could be established because all randomized trials had follow‐up of 12 months or less. These findings support a prudent and context‐specific application of nicotinamide in high‐risk immunocompetent adults, whereas in transplant recipients larger and longer studies are needed before its routine use as secondary prevention can be recommended.

## Author Contributions

Andrea Lizeth Guerra Jiménez: conceptualization; search strategy development; literature searching and investigation; study selection; data extraction and validation; risk‐of‐bias assessment; interpretation of findings; writing–original draft; and writing–review and editing.

Juan Carlos Ortegón Garciaherreros: conceptualization; search strategy development; literature searching and investigation; study selection; data extraction and validation; risk‐of‐bias assessment; interpretation of findings; writing–original draft; and writing–review and editing.

Lilia Patricia Suárez Escobar: conceptualization; search strategy development; literature searching and investigation; study selection; data extraction and validation; risk‐of‐bias assessment; interpretation of findings; writing–original draft; and writing–review and editing.

Laura Valentina Vargas Monroy: conceptualization; search strategy development; literature searching and investigation; study selection; data extraction and validation; risk‐of‐bias assessment; interpretation of findings; writing–original draft; and writing–review and editing.

María Camila Madariaga Estupiñán: conceptualization; methodology; search strategy development; literature searching and investigation; study selection; data extraction, validation, and data curation; risk‐of‐bias assessment; formal statistical analysis and meta‐analysis; GRADE certainty assessment; visualization; interpretation of findings; project administration; writing–original draft; and writing–review and editing.

All authors contributed to all stages of the review.

## Funding

This research did not receive any specific grant from funding agencies in the public, commercial, or not‐for‐profit sectors.

## Disclosure

All authors approved the final version of the manuscript and agreed to be accountable for the integrity of the work.

## Ethics Statement

This study was registered under CRD420261407650 (Version 1.0) on the International Prospective Register of Systematic Reviews (PROSPERO), National Institute for Health and Care Research, University of York. The full record is publicly available at https://www.crd.york.ac.uk/prospero/ using the registration identifier above.

## Conflicts of Interest

The authors declare no conflicts of interest.

## Supporting Information

Additional supporting information can be found online in the Supporting Information section.

## Supporting information


**Supporting Information** The Supporting Methods contain the complete electronic search strategies. Supporting Tables 1–7 present the full‐text exclusions, outcome‐specific RoB 2 judgments (Table 2) and the ROBINS‐I assessment of the nonrandomized study (Table 2B), the SWiM grouping matrix, the meta‐analysis dataset and derivations including the estimator‐sensitivity comparison (Table 4C), the GRADE profile, the planned subgroup and sensitivity analyses (Table 6), and the consolidated adverse‐event table (Table 7). Supporting Figures 1–4 present the exploratory and sensitivity forest plots. The completed PRISMA 2020 checklist is included in the Supporting Information.

## Data Availability

The data that support the findings of this study are available from the corresponding author upon reasonable request.
