# Supplementary material for: Oral Nicotinamide for the Secondary Prevention of Cutaneous Squamous Cell Carcinoma and Other Keratinocyte Carcinomas: A Systematic Review
Source: J Skin Cancer. 2026 Oct 5;2026:8756577. doi: 10.1155/jskc/8756577 (PMC13635799; doi:10.1155/jskc/8756577)
Supplement: Supplementary file 1 [file JSKC-2026-8756577-s001.docx]

**Supplementary material**

**Oral nicotinamide for the secondary prevention of cutaneous squamous cell carcinoma and other keratinocyte carcinomas**

# Contents

- Supplementary Methods. Complete electronic search strategies
- Supplementary Table 1. Reports excluded at full text and reasons for exclusion
- Supplementary Tables 2 and 2B. Outcome-specific RoB 2 judgments and ROBINS-I assessment of the non-randomized study
- Supplementary Table 3. SWiM grouping and synthesis feasibility matrix
- Supplementary Tables 4A-4C. Meta-analysis dataset, derivations, and estimator-sensitivity analyses
- Supplementary Table 5. Detailed GRADE profile
- Supplementary Table 6. Planned subgroup and sensitivity analyses
- Supplementary Table 7. Consolidated adverse events by treatment group
- Supplementary Figures 1-4. Exploratory and sensitivity forest plots
- Completed PRISMA 2020 checklist

# Supplementary Methods. Complete electronic search strategies

The searches covered each database from inception through May 30, 2026. No language restrictions or study-design filters were applied. The search retrieved 1,076 records before deduplication: PubMed/MEDLINE, 146; Embase, 828; and LILACS through the Virtual Health Library, 102.

## PubMed/MEDLINE

*Platform: PubMed. Last searched: May 30, 2026. Coverage: database inception to May 30, 2026. Filters/limits: none. Records retrieved: 146.*

```
(
  "Niacinamide"[Mesh]
  OR nicotinamide*[tiab]
  OR niacinamide*[tiab]
  OR "niacin amide"[tiab]
  OR "nicotinic acid amide"[tiab]
  OR "vitamin B3"[tiab]
)
AND
(
  "Skin Neoplasms"[Mesh]
  OR "Carcinoma, Squamous Cell"[Mesh]
  OR "Carcinoma, Basal Cell"[Mesh]
  OR "Keratosis, Actinic"[Mesh]
  OR "nonmelanoma skin cancer*"[tiab]
  OR "non-melanoma skin cancer*"[tiab]
  OR "keratinocyte carcinoma*"[tiab]
  OR "cutaneous squamous cell carcinoma*"[tiab]
  OR cSCC[tiab]
  OR SCC[tiab]
  OR "squamous cell carcinoma*"[tiab]
  OR "basal cell carcinoma*"[tiab]
  OR BCC[tiab]
  OR "actinic keratos*"[tiab]
)
```

## Embase

*Platform: Embase.com. Last searched: May 30, 2026. Coverage: database inception to May 30, 2026. Filters/limits: none. Records retrieved: 828.*

```
(
  'nicotinamide'/exp
  OR nicotinamide*:ti,ab,kw
  OR niacinamide*:ti,ab,kw
  OR 'niacin amide':ti,ab,kw
  OR 'nicotinic acid amide':ti,ab,kw
  OR 'vitamin b3':ti,ab,kw
)
AND
(
  'skin tumor'/exp
  OR 'non melanoma skin cancer'/exp
  OR 'squamous cell carcinoma'/exp
  OR 'basal cell carcinoma'/exp
  OR 'actinic keratosis'/exp
  OR 'nonmelanoma skin cancer*':ti,ab,kw
  OR 'non-melanoma skin cancer*':ti,ab,kw
  OR 'keratinocyte carcinoma*':ti,ab,kw
  OR 'cutaneous squamous cell carcinoma*':ti,ab,kw
  OR cscc:ti,ab,kw
  OR scc:ti,ab,kw
  OR 'squamous cell carcinoma*':ti,ab,kw
  OR 'basal cell carcinoma*':ti,ab,kw
  OR bcc:ti,ab,kw
  OR 'actinic keratos*':ti,ab,kw
)
```

## LILACS through the Virtual Health Library (VHL/BVS)

*Platform: Virtual Health Library (VHL/BVS), LILACS database. Last searched: May 30, 2026. Coverage: database inception to May 30, 2026. Filters/limits: none. Records retrieved: 102.*

```
(
  nicotinamida
  OR niacinamida
  OR nicotinamide
  OR niacinamide
  OR "vitamina B3"
  OR "vitamin B3"
)
AND
(
  "carcinoma escamocelular cutáneo"
  OR "carcinoma espinocelular"
  OR "carcinoma de células escamosas"
  OR "cutaneous squamous cell carcinoma"
  OR cSCC
  OR "cáncer de piel no melanoma"
  OR "cancer de piel no melanoma"
  OR "non-melanoma skin cancer"
  OR "nonmelanoma skin cancer"
  OR "carcinoma queratinocítico"
  OR "keratinocyte carcinoma"
  OR "carcinoma basocelular"
  OR "basal cell carcinoma"
  OR "queratosis actínica"
  OR "queratosis actinica"
  OR "actinic keratosis"
)
```

# Supplementary Table 1. Reports excluded at full text and reasons for exclusion

| **No.** | **Author/year** | **Abbreviated title** | **Journal** | **DOI/PMID** | **Reason for exclusion** | **Category** |
| --- | --- | --- | --- | --- | --- | --- |
| 1 | Gilmore, 2018 | Nicotinamide and skin cancer chemoprevention… | Australas J Dermatol | DOI not reported | Narrative review without original data | Ineligible design |
| 2 | Snaidr et al., 2019 | Nicotinamide for photoprotection and skin cancer… | Exp Dermatol | 10.1111/exd.13819 | Review; no original primary data | Ineligible design |
| 3 | Tosti et al., 2024 | The role of nicotinamide as chemo-preventive agent… | Nutrients | 10.3390/nu16010100 | Prior systematic review and meta-analysis; not a primary study | Ineligible design |
| 4 | Drago et al., 2016 | Nicotinamide for skin-cancer chemoprevention (letter) | NEJM | 10.1056/NEJMc1601694 | Letter/comment without independent original data | Ineligible design |
| 5 | Drago et al., 2017 | Prevention of NMSC with nicotinamide in transplant… | Eur J Dermatol | 10.1684/ejd.2017.3029 | Population and outcomes centered mainly on actinic keratoses; the secondary-prevention KC/NMSC population was not clearly aligned, and data partially overlapped with Chen 2016. | Ineligible population/outcome; overlap |
| 6 | Surjana et al., 2012 | Oral nicotinamide reduces actinic keratoses (phase II) | J Invest Dermatol | 10.1038/jid.2011.459 | Population without prior history of KC/NMSC (primary prevention of AK) | Ineligible population |
| 7 | Desai et al., 2021 | Nicotinamide for KC chemoprevention: survey of Mohs… | Dermatol Surg | 10.1097/DSS.0000000000002806 | Practice survey; no patient outcome data | Ineligible design |
| 8 | Gollins et al., 2020 | Feasibility of nicotinamide trial in OTR UK | Br J Dermatol | 10.1111/bjd.19038 | Feasibility study; no efficacy data on KC outcomes | Ineligible design |
| 9 | Yelamos et al., 2017 | Reply: phase II RCT nicotinamide renal transplant | Br J Dermatol | 10.1111/bjd.15171 | Comment/letter without original data | Ineligible design |
| 10 | Zhao, 2016 | Nicotinamide for skin-cancer chemoprevention (letter) | NEJM | 10.1056/NEJMc1514791 | Letter without original data | Ineligible design |
| 11 | Schmults et al., 2023 | Comment on ONTRANS (NEJM letter) | NEJM | 10.1056/NEJMc2303841 | Comment/letter without original data | Ineligible design |
| 12 | Hwang et al., 2025 | Nicotinamide for secondary KC prevention in SOTR | Arch Dermatol Res | 10.1007/s00403-025-04296-7 | Two reasons: (1) the reported age range included participants younger than 18 years without separable adult data; and (2) the retrospective before-after design lacked a concurrent external comparator, which was required for non-randomized studies. | Ineligible population and design |

# Supplementary Table 2. Outcome-specific RoB 2 risk-of-bias judgments

RoB 2 judgments refer to the effect of assignment to intervention. For ONTRAC, ONTRANS, and Chen 2016, the evaluated outcome was total KC/NMSC during the intervention period; for Zhang 2023, it was actinic keratosis count/tolerability. Two reviewers assessed each result independently, with disagreements resolved by consensus or third-reviewer adjudication.

| **Study** | **Outcome/estimand** | **Domain** | **Judgment** | **Rationale** |
| --- | --- | --- | --- | --- |
| Chen et al., 2015 / ONTRAC4 | New total NMSC during 12 months; assignment effect | D1: bias arising from the randomization process | Low | Centralized randomization and comparable groups. |
| Chen et al., 2015 / ONTRAC4 | New total NMSC during 12 months; assignment effect | D2: bias due to deviations from intended interventions | Low | Double-blind, placebo-controlled design with adequate adherence. |
| Chen et al., 2015 / ONTRAC4 | New total NMSC during 12 months; assignment effect | D3: bias due to missing outcome data | Low | Adequate follow-up and outcome data. |
| Chen et al., 2015 / ONTRAC4 | New total NMSC during 12 months; assignment effect | D4: bias in outcome measurement | Low | Blinded clinical and histopathologic assessment. |
| Chen et al., 2015 / ONTRAC4 | New total NMSC during 12 months; assignment effect | D5: bias in selection of the reported result | Low | No evidence of selective reporting was identified; the primary tumor-count outcomes described in the trial report were reported. |
| Chen et al., 2015 / ONTRAC4 | New total NMSC during 12 months; assignment effect | Overall judgment | Low | No domains with relevant concerns. |
| Allen et al., 2023 / ONTRANS5 | New total KC during 12 months; assignment effect | D1: bias arising from the randomization process | Low | Randomized, double-blind trial. |
| Allen et al., 2023 / ONTRANS5 | New total KC during 12 months; assignment effect | D2: bias due to deviations from intended interventions | Low | Intervention and placebo comparator; deviations not suggestive of important bias. |
| Allen et al., 2023 / ONTRANS5 | New total KC during 12 months; assignment effect | D3: bias due to missing outcome data | Some concerns | Modified intention-to-treat analysis and incomplete follow-up in some participants. |
| Allen et al., 2023 / ONTRANS5 | New total KC during 12 months; assignment effect | D4: bias in outcome measurement | Low | Adequate, blinded outcome measurement. |
| Allen et al., 2023 / ONTRANS5 | New total KC during 12 months; assignment effect | D5: bias in selection of the reported result | Low | No evidence of selective reporting was identified for the principal KC outcomes in the available trial report. |
| Allen et al., 2023 / ONTRANS5 | New total KC during 12 months; assignment effect | Overall judgment | Some concerns | Overall judgment driven by concerns about missing data/follow-up. |
| Chen et al., 201611 | New total NMSC during 6 months; assignment effect | D1: bias arising from the randomization process | Low | Random allocation and placebo-controlled design. |
| Chen et al., 201611 | New total NMSC during 6 months; assignment effect | D2: bias due to deviations from intended interventions | Low | Double-blind, placebo comparator. |
| Chen et al., 201611 | New total NMSC during 6 months; assignment effect | D3: bias due to missing outcome data | Low | Outcome data sufficient for the reported analysis. |
| Chen et al., 201611 | New total NMSC during 6 months; assignment effect | D4: bias in outcome measurement | Low | Blinded dermatologic and histopathologic assessment. |
| Chen et al., 201611 | New total NMSC during 6 months; assignment effect | D5: bias in selection of the reported result | Low | No evidence of selective reporting was identified for the principal NMSC outcomes in the available trial report. |
| Chen et al., 201611 | New total NMSC during 6 months; assignment effect | Overall judgment | Low | The small size affects precision, not internal bias. |
| Zhang et al., 202312 | AK count/tolerability at 12 months; assignment effect | D1: bias arising from the randomization process | Low | Randomized, double-blind trial. |
| Zhang et al., 202312 | AK count/tolerability at 12 months; assignment effect | D2: bias due to deviations from intended interventions | High | Relevant dose modification during the study due to gastrointestinal events. |
| Zhang et al., 202312 | AK count/tolerability at 12 months; assignment effect | D3: bias due to missing outcome data | High | Important differential losses: 40% in the nicotinamide group and 0% in placebo. |
| Zhang et al., 202312 | AK count/tolerability at 12 months; assignment effect | D4: bias in outcome measurement | Low | Clinical outcome measurement without a clear signal of differential bias. |
| Zhang et al., 202312 | AK count/tolerability at 12 months; assignment effect | D5: bias in selection of the reported result | Some concerns | Limited reporting and the small pilot sample left uncertainty about selective reporting. |
| Zhang et al., 202312 | AK count/tolerability at 12 months; assignment effect | Overall judgment | High | Overall judgment driven by dose deviations and differential losses. |

# Supplementary Table 2B. ROBINS-I risk-of-bias assessment of the included non-randomized study

Assessment refers to the effect of starting versus not starting oral nicotinamide on time to the next skin cancer. The target-trial framing compared initiation of nicotinamide 500 mg twice daily with no initiation in adults with a prior skin cancer.

| **Study** | **ROBINS-I domain** | **Judgment** | **Rationale** |
| --- | --- | --- | --- |
| Breglio et al., 2025 [24] | D1. Confounding | Serious | Propensity-score matching on the number and timing of prior skin cancers, age, sex, race, acitretin exposure, field therapy, chronic lymphocytic leukaemia, and transplant status (absolute standardized mean differences <0.1 for most strata), but matching balance failed for patients treated after nine or more prior skin cancers (ASMD 0.11-0.20). Residual and unmeasured confounding (sun exposure, skin phototype, surveillance intensity) remained, as acknowledged by the authors. Nicotinamide-exposed patients had more higher-risk features (chronic lymphocytic leukaemia, acitretin exposure, transplant), a confounding-by-indication that would bias toward the null. |
| Breglio et al., 2025 [24] | D2. Selection of participants | Moderate | Cohort assembled from a defined health-system population with a prior skin cancer; exclusion of skin cancers treated within 90 days of baseline introduces a degree of immortal-time bias by design. |
| Breglio et al., 2025 [24] | D3. Classification of interventions | Serious | Nicotinamide exposure ascertained from prescription/pharmacy records; over-the-counter or non-Veterans-Affairs nicotinamide use in the comparator group could not be captured, allowing exposure misclassification; the authors explicitly note this may have underestimated the true effect (bias toward the null). |
| Breglio et al., 2025 [24] | D4. Deviations from intended interventions | Moderate | Real-world adherence and discontinuation not fully observable; new-user design with a minimum 30-day exposure mitigates but does not eliminate this concern. |
| Breglio et al., 2025 [24] | D5. Missing data | Moderate | Administrative dataset with largely complete outcome capture within the system; out-of-system events not observable. |
| Breglio et al., 2025 [24] | D6. Measurement of outcomes | Moderate | Skin cancers identified from ICD/CPT codes rather than centralized histology; outcome ascertainment independent of exposure status. |
| Breglio et al., 2025 [24] | D7. Selection of the reported result | Low | Pre-specified primary and subgroup analyses reported; a published correction to Figure 4 (October 15, 2025) was accounted for by using the corrected values. |
| Breglio et al., 2025 [24] | Overall | Serious | Driven by confounding and intervention classification. Several biases were expected by the authors to attenuate the estimate toward the null, so the observed protective associations are unlikely to be artefacts of these limitations. |

# Supplementary Table 3. SWiM grouping and synthesis feasibility matrix

| **Grouping dimension** | **Planned categories** | **Feasibility in this review** | **How it was handled** |
| --- | --- | --- | --- |
| Study design | RCTs vs non-randomized/cohorts | Yes | Four randomized trials and one retrospective cohort were included. Randomized and observational designs were synthesized separately; the cohort was not statistically pooled with the trials. |
| Immune status | Immunocompetent vs transplant recipients or other immunosuppressed | Yes | Reported separately; the main exploratory meta-analysis was restricted to transplant recipients. |
| Prior skin cancer history | cSCC only vs any KC/NMSC | Partial | Most studies included a history of KC/NMSC; described narratively. |
| Nicotinamide dose | 500 mg twice daily vs other regimens | Partial | Comparable trials used 500 mg twice daily; Zhang had dose modifications for tolerability. |
| Comparator | Placebo vs active comparator/usual care | Yes | Randomized trials used placebo comparators; the retrospective cohort used propensity-score matched unexposed patients. |
| Outcome type | cSCC, total KC/NMSC, BCC, AK, safety | Yes | Outcomes were presented separately in tables and meta-analyses when possible. |
| Follow-up | ≤12 months vs >12 months | Yes, descriptively | All randomized trials had follow-up ≤12 months; the retrospective cohort contributed longer-term observational follow-up. Duration was described rather than pooled across designs. |
| Vote counting by direction of effect | Exploratory when pooling was not possible | Yes | Avoided as a main conclusion; narrative synthesis by outcome and population was used. |

# Supplementary Table 4. Dataset and derivations used for the meta-analyses

Tumor counts are numbers of lesions, not numbers of participants with at least one event. All pooled estimates were rate ratios. For Chen 2016 cSCC/SCC and BCC, the rate ratio was reconstructed from the reported relative rate difference (RRD) as RR = 1 - RRD; the confidence interval was transformed as [1 - upper RRD limit, 1 - lower RRD limit]. Log effects and standard errors were calculated from the rate-ratio confidence intervals.

**Supplementary Table 4A.** Clinical data and effect estimates

| **Analysis/outcome** | **Study** | **Population** | **Follow-up** | **NAM lesions** | **Placebo lesions** | **Effect measure** | **Estimate** | **95% CI** |
| --- | --- | --- | --- | --- | --- | --- | --- | --- |
| Total KC/NMSC – transplant | Allen 2023 / ONTRANS | Solid organ transplant recipients | 12 months | 207 | 210 | Rate ratio | 1.0 | 0.80–1.30 |
| Total KC/NMSC – transplant | Chen 2016 phase II | Renal transplant recipients | 6 months | 30 | 45 | Rate ratio | 0.65 | 0.30–1.60 |
| cSCC/SCC – transplant | Allen 2023 / ONTRANS | Solid organ transplant recipients | 12 months | 138 | 151 | Rate ratio | 0.9 | 0.60–1.20 |
| cSCC/SCC – transplant | Chen 2016 phase II | Renal transplant recipients | 6 months | 23 | 24 | Rate ratio | 0.93 | 0.35–2.51 |
| BCC – transplant | Allen 2023 / ONTRANS | Solid organ transplant recipients | 12 months | 69 | 59 | Rate ratio | 1.4 | 0.80–2.30 |
| BCC – transplant | Chen 2016 phase II | Renal transplant recipients | 6 months | 7 | 21 | Rate ratio | 0.33 | 0.08–1.45 |
| Total KC/NMSC – all populations | Chen 2015 / ONTRAC | High-risk immunocompetent | 12 months | 336 | 463 | Rate ratio | 0.77 | 0.62–0.96 |
| Total KC/NMSC – all populations | Allen 2023 / ONTRANS | Solid organ transplant recipients | 12 months | 207 | 210 | Rate ratio | 1.0 | 0.80–1.30 |
| Total KC/NMSC – all populations | Chen 2016 phase II | Renal transplant recipients | 6 months | 30 | 45 | Rate ratio | 0.65 | 0.30–1.60 |
| cSCC/SCC – all populations | Chen 2015 / ONTRAC | High-risk immunocompetent | 12 months | 97 | 136 | Rate ratio | 0.7 | 0.49–1.00 |
| cSCC/SCC – all populations | Allen 2023 / ONTRANS | Solid organ transplant recipients | 12 months | 138 | 151 | Rate ratio | 0.9 | 0.60–1.20 |
| cSCC/SCC – all populations | Chen 2016 phase II | Renal transplant recipients | 6 months | 23 | 24 | Rate ratio | 0.93 | 0.35–2.51 |

**Supplementary Table 4B.** Effect derivation and log-scale data

| **Analysis/outcome** | **Study** | **Source/derivation** | **Log effect** | **SE** |
| --- | --- | --- | --- | --- |
| Total KC/NMSC – transplant | Allen 2023 / ONTRANS | Rate ratio and 95% CI reported directly in the trial report. | 0.0 | 0.124 |
| Total KC/NMSC – transplant | Chen 2016 phase II | Rate ratio and 95% CI reported directly in the trial report. | -0.431 | 0.427 |
| cSCC/SCC – transplant | Allen 2023 / ONTRANS | Rate ratio and 95% CI reported directly in the trial report. | -0.105 | 0.177 |
| cSCC/SCC – transplant | Chen 2016 phase II | Reconstructed from reported negative-binomial RRD: RR = 1 - RRD; CI transformed from RRD limits. | -0.073 | 0.503 |
| BCC – transplant | Allen 2023 / ONTRANS | Rate ratio and 95% CI reported directly in the trial report. | 0.336 | 0.269 |
| BCC – transplant | Chen 2016 phase II | Reconstructed from reported negative-binomial RRD: RR = 1 - RRD; CI transformed from RRD limits. | -1.109 | 0.739 |
| Total KC/NMSC – all populations | Chen 2015 / ONTRAC | Rate ratio and 95% CI reported directly in the trial report. | -0.261 | 0.112 |
| Total KC/NMSC – all populations | Allen 2023 / ONTRANS | Rate ratio and 95% CI reported directly in the trial report. | 0.0 | 0.124 |
| Total KC/NMSC – all populations | Chen 2016 phase II | Rate ratio and 95% CI reported directly in the trial report. | -0.431 | 0.427 |
| cSCC/SCC – all populations | Chen 2015 / ONTRAC | Rate ratio and 95% CI reported directly in the trial report. | -0.357 | 0.182 |
| cSCC/SCC – all populations | Allen 2023 / ONTRANS | Rate ratio and 95% CI reported directly in the trial report. | -0.105 | 0.177 |
| cSCC/SCC – all populations | Chen 2016 phase II | Reconstructed from reported negative-binomial RRD: RR = 1 - RRD; CI transformed from RRD limits. | -0.073 | 0.503 |

# Supplementary Table 4C. Sensitivity of pooled estimates to the choice of heterogeneity estimator

Random-effects pooling of rate ratios on the log scale. DL, DerSimonian-Laird; REML, restricted maximum likelihood; HKSJ, Hartung-Knapp-Sidik-Jonkman. All analyses include randomized trials only; the observational cohort was not pooled. Values below 1 favor nicotinamide. These estimator-sensitivity analyses were added during revision as post hoc robustness checks and were not pre-specified in the protocol.

| **Analysis (population)** | **Studies (k)** | **I²; τ²** | **DL — RR (95% CI)** | **REML — RR (95% CI)** | **HKSJ — RR (95% CI)** |
| --- | --- | --- | --- | --- | --- |
| Total KC/NMSC (transplant; main) | 2 | 0.0%; 0.000 | 0.97 (0.77–1.22) | 0.97 (0.77–1.22) | 0.97 (0.22–4.19) |
| cSCC/SCC (transplant) | 2 | 0.0%; 0.000 | 0.90 (0.65–1.25) | 0.90 (0.65–1.25) | 0.90 (0.80–1.03) |
| BCC (transplant) | 2 | 70.4%; 0.735 | 0.80 (0.20–3.18) | 0.80 (0.20–3.18) | 0.80 (≈0.0001–6113) |
| Total KC/NMSC (all populations) | 3 | 30.5%; 0.011–0.016 | 0.86 (0.69–1.06) | 0.85 (0.68–1.08) | 0.85 (0.55–1.33) |
| cSCC/SCC (all populations) | 3 | 0.0%; 0.000 | 0.80 (0.63–1.02) | 0.80 (0.63–1.02) | 0.80 (0.55–1.18) |

* For BCC in transplant recipients, the classical HKSJ interval is mathematically estimable but extremely wide and clinically uninformative in this two-study, high-heterogeneity configuration.

Interpretation. Point estimates were essentially invariant across estimators, and the qualitative conclusion of no demonstrated benefit in transplant recipients was unchanged. DL and REML coincided or were nearly identical when between-study variance was estimated as zero. With only two studies, however, classical HKSJ behavior was unstable: it markedly widened the main total KC/NMSC interval, produced a narrower interval for cSCC/SCC because residual heterogeneity was nearly zero, and yielded an extremely wide, uninformative interval for BCC. Accordingly, HKSJ was treated as a small-sample robustness check rather than as evidence of greater precision.

# Supplementary Table 5. Detailed GRADE profile

| **Outcome** | **Population** | **Risk of bias** | **Inconsistency** | **Indirectness** | **Imprecision** | **Publication bias** | **Final certainty** | **Explanation** |
| --- | --- | --- | --- | --- | --- | --- | --- | --- |
| Total KC/NMSC | Transplant recipients | Serious | Not serious | Not serious | Serious | Not assessable | Low | The dominant study had some concerns, and the CI includes both benefit and no effect. |
| cSCC/SCC | Transplant recipients | Not serious to serious | Not serious | Not serious | Serious | Not assessable | Low | Wide CI; one estimate from the small trial was approximated from the reported data. |
| BCC | Transplant recipients | Serious | Serious | Not serious | Very serious | Not assessable | Very low | Very wide CI and high heterogeneity; effect compatible with benefit or harm. |
| Total KC/NMSC | All populations | Serious | Not serious | Serious | Serious | Not assessable | Very low | Sensitivity analysis mixing clinically heterogeneous populations. |
| cSCC/SCC | All populations | Serious | Not serious | Serious | Serious | Not assessable | Very low | Sensitivity analysis mixing immunocompetent and transplant recipients. |
| Any skin cancer / cSCC | Overall cohort (observational) | Serious | Not assessable (single cohort) | Not serious | Not serious | Not assessable | Very low | Observational evidence starts at low certainty and was downgraded for serious risk of bias, mainly residual confounding and exposure classification; findings are supportive, not confirmatory. |
| Actinic keratoses | Immunocompetent and transplant | Serious | Serious | Serious | Serious | Not assessable | Low to very low | Variable results across populations and a pilot trial with high risk of bias. |
| Adverse events/tolerability | All included populations | Serious | Not serious | Not serious | Serious | Not assessable | Low | Few studies and heterogeneous safety reporting; ONTRAC showed an infection signal, ONTRANS showed no excess of serious infection events, and one pilot trial showed gastrointestinal tolerability problems and differential losses. AE-related discontinuation was not reported by arm for all trials. |

# Supplementary Table 6. Planned subgroup and sensitivity analyses

| **Planned analysis** | **Status** | **How it was handled / reason** |
| --- | --- | --- |
| Immunocompetent vs transplant recipients or other immunosuppressed | Implemented | Reported separately; the main exploratory meta-analysis was restricted to transplant recipients. |
| History of cSCC vs history of any KC/NMSC | Not formally feasible | Studies used prior KC/NMSC history criteria; insufficient studies for separate subgroups. |
| Nicotinamide 500 mg twice daily vs other regimens | Partial | Comparable trials used 500 mg twice daily; Zhang modified the dose for tolerability. |
| Initiation after first skin cancer vs multiple prior cancers | Partially informative (observational only) | Breglio et al. reported the strongest association when nicotinamide was initiated after the first skin cancer, with attenuation after subsequent cancers. This timing analysis was treated as hypothesis-generating and was not pooled with randomized evidence. |
| Follow-up ≤12 months vs >12 months | Not formally pooled | All randomized trials had follow-up ≤12 months; the retrospective cohort provided longer observational follow-up. Cross-design pooling by duration was not performed. |
| Randomized trials vs cohorts | Implemented descriptively | Four randomized trials and one retrospective cohort were included. Designs were kept separate; the cohort was synthesized narratively and assessed with ROBINS-I. |
| Exclusion of studies at high risk of bias | Does not change the main analysis | The high-risk-of-bias trial was not included in the main meta-analysis. |
| Use of adjusted observational estimates | Implemented narratively | Adjusted hazard ratios from Breglio et al. were extracted and synthesized narratively; they were not pooled with randomized rate ratios. |
| Exclusion of studies with an unclear route of administration | Applied at eligibility | Studies without confirmation of oral nicotinamide were excluded. |
| Exclusion of conference abstracts or abstract-only studies | Applied | Abstracts and registries without full text/results were not included. |
| Publication bias and small-study effects | Narrative | Funnel plots and formal tests were not performed because k < 10 for all outcomes. |

# Supplementary Table 7. Consolidated adverse events by treatment group

Data are presented as reported in the primary publications and, for ONTRANS, in the supplementary safety data of the original ONTRANS publication [5]. Numerical values are shown only when directly reported or when a published percentage is presented with its denominator. NR indicates that the specific treatment-arm outcome was not reported; no adverse-event values were imputed.

| **Study (population)** | **Adverse-event measure** | **Nicotinamide** | **Placebo** | **Notes** |
| --- | --- | --- | --- | --- |
| Chen 2015 / ONTRAC (immunocompetent) [4] | Participants per arm | 193 | 193 | 500 mg twice daily; 12 months. |
| Chen 2015 / ONTRAC [4] | Overall/serious AE profile | NR | NR | No significant between-group difference in the number or type of adverse events was reported; arm-level overall AE/SAE totals were not available in the primary trial text used for extraction. |
| Chen 2015 / ONTRAC [4] | Gastrointestinal events | NR | NR | No specific arm-level gastrointestinal-event count was available in the primary trial text used for extraction. |
| Chen 2015 / ONTRAC [4] | Total infections (n) | 90 | 73 | Not statistically significant. |
| Chen 2015 / ONTRAC [4] | Grade 3–5 infections (n) | 10 | 5 | Not statistically significant. |
| Chen 2015 / ONTRAC [4] | Skin infections (n) | 14 | 4 | Statistically significant (P < .05). |
| Allen 2023 / ONTRANS (transplant) [5] | Safety-analysis population | 78 | 79 | One participant randomized to nicotinamide received no study treatment and was excluded from the safety set (ONTRANS original publication and supplementary materials). |
| Allen 2023 / ONTRANS [5] | Participants with ≥1 adverse event | 59/78 (75.6%) | 54/79 (68.4%) | Derived from the ONTRANS supplementary safety data; worst adverse-event grade per participant. |
| Allen 2023 / ONTRANS [5] | Total adverse-event episodes | 136 | 150 | ONTRANS supplementary safety data; counts of adverse events by grade. |
| Allen 2023 / ONTRANS [5] | Serious adverse-event episodes | 38 | 35 | Derived from the ONTRANS supplementary safety data; counts of serious adverse events by grade. These are event counts, not numbers of participants with ≥1 SAE. |
| Allen 2023 / ONTRANS [5] | Participants with gastrointestinal adverse events | 15.4% | 16.5% | Derived from the ONTRANS supplementary safety data; percentage of participants by gastrointestinal class and worst grade. |
| Allen 2023 / ONTRANS [5] | Gastrointestinal adverse-event episodes | 13 | 17 | Derived from the ONTRANS supplementary safety data; all grades. |
| Allen 2023 / ONTRANS [5] | Serious gastrointestinal adverse-event episodes | 3 | 3 | Derived from the ONTRANS supplementary safety data. |
| Allen 2023 / ONTRANS [5] | Infection/infestation adverse-event episodes | 39 | 36 | Derived from the ONTRANS supplementary safety data; all grades. |
| Allen 2023 / ONTRANS [5] | Serious infection/infestation episodes | 10 | 11 | Derived from the ONTRANS supplementary safety data; no numerical excess of serious infections with nicotinamide. |
| Allen 2023 / ONTRANS [5] | Withdrawal/discontinuation due to adverse event | NR | NR | Not reported by treatment arm in the primary article or the trial's supplementary materials; not inferred from overall withdrawals or adherence. |
| Chen 2016 (phase II, renal transplant) [11] | Safety concerns | None reported | None reported | Small sample; no significant increase in adverse effects or meaningful laboratory/blood-pressure differences was reported. |
| Zhang 2023 (pilot, renal transplant) [12] | Gastrointestinal intolerance | Present; prompted dose reduction | Not reported to same degree | Gastrointestinal tolerability problems led to protocol dose modification. |
| Zhang 2023 [12] | Treatment discontinuation / differential losses | ~40% | 0% | Substantial differential loss in the nicotinamide arm; exact attribution to adverse events was incompletely reported. |

Note. For ONTRANS, the safety-analysis population was 78 participants receiving nicotinamide and 79 receiving placebo because one participant randomized to nicotinamide received no study treatment. The original ONTRANS supplementary materials provide arm-level adverse-event data, including overall adverse events, serious adverse events, gastrointestinal events, and infections. Withdrawal or treatment discontinuation specifically due to adverse events was not reported by treatment arm in the article or the trial's supplementary materials and is therefore marked NR.

# Supplementary Figures

Supplementary Figure 1. Forest plot of oral nicotinamide versus placebo for cSCC/SCC in transplant recipients. Exploratory random-effects analysis of two randomized trials (180 participants total). Lesion counts (nicotinamide vs placebo) were 138 vs 151 in ONTRANS and 23 vs 24 in Chen 2016; study weights were 89.0% and 11.0%, respectively. The pooled rate ratio was 0.90 (95% CI, 0.65–1.25). The Chen 2016 estimate was reconstructed from the reported relative rate difference.

Supplementary Figure 2. Forest plot of oral nicotinamide versus placebo for BCC in transplant recipients. Exploratory random-effects analysis of two randomized trials (180 participants total). Lesion counts (nicotinamide vs placebo) were 69 vs 59 in ONTRANS and 7 vs 21 in Chen 2016; study weights were 61.3% and 38.7%, respectively. The pooled rate ratio was 0.80 (95% CI, 0.20–3.18; I² = 70.4%). This was a non-primary analysis owing to imprecision and heterogeneity.

Supplementary Figure 3. Sensitivity analysis of oral nicotinamide versus placebo for total KC/NMSC across all randomized populations. Three trials contributed 566 participants. Lesion counts (nicotinamide vs placebo) were 336 vs 463 in ONTRAC, 207 vs 210 in ONTRANS, and 30 vs 45 in Chen 2016; study weights were 49.7%, 44.3%, and 6.1%, respectively. The pooled rate ratio was 0.86 (95% CI, 0.69–1.06). This analysis was not considered a main analysis owing to clinical heterogeneity between populations.

Supplementary Figure 4. Sensitivity analysis of oral nicotinamide versus placebo for cSCC/SCC across all randomized populations. Three trials contributed 566 participants. Lesion counts (nicotinamide vs placebo) were 97 vs 136 in ONTRAC, 138 vs 151 in ONTRANS, and 23 vs 24 in Chen 2016; study weights were 45.7%, 48.4%, and 6.0%, respectively. The pooled rate ratio was 0.80 (95% CI, 0.63–1.02). This analysis was not considered a main analysis owing to clinical heterogeneity between populations.

# PRISMA 2020 checklist

*Completed PRISMA 2020 checklist. Locations refer to sections and subsections of the main manuscript unless a supplementary item is named. Item wording follows the PRISMA 2020 statement.*

*From: Page MJ, McKenzie JE, Bossuyt PM, Boutron I, Hoffmann TC, Mulrow CD, et al. The PRISMA 2020 statement: an updated guideline for reporting systematic reviews. BMJ. 2021;372:n71. doi:10.1136/bmj.n71.*

| **Section and topic** | **Item #** | **Checklist item** | **Location where item is reported** |
| --- | --- | --- | --- |
| **TITLE** | | | |
| Title | 1 | Identify the report as a systematic review. | Title |
| **ABSTRACT** | | | |
| Abstract | 2 | See the PRISMA 2020 for Abstracts checklist. | Abstract (structured abstract) |
| **INTRODUCTION** | | | |
| Rationale | 3 | Describe the rationale for the review in the context of existing knowledge. | Introduction |
| Objectives | 4 | Provide an explicit statement of the objective(s) or question(s) the review addresses. | Abstract (Objective); Introduction (final paragraph) |
| **METHODS** | | | |
| Eligibility criteria | 5 | Specify the inclusion and exclusion criteria for the review and how studies were grouped for the syntheses. | Methods, Eligibility criteria |
| Information sources | 6 | Specify all databases, registers, websites, organisations, reference lists and other sources searched or consulted to identify studies. Specify the date when each source was last searched or consulted. | Methods, Information sources and search strategy; Supplementary Methods |
| Search strategy | 7 | Present the full search strategies for all databases, registers and websites, including any filters and limits used. | Supplementary Methods (full search strategies); Methods, Information sources and search strategy |
| Selection process | 8 | Specify the methods used to decide whether a study met the inclusion criteria of the review, including how many reviewers screened each record and each report retrieved, whether they worked independently, and if applicable, details of automation tools used in the process. | Methods, Study selection |
| Data collection process | 9 | Specify the methods used to collect data from reports, including how many reviewers collected data from each report, whether they worked independently, any processes for obtaining or confirming data from study investigators, and if applicable, details of automation tools used in the process. | Methods, Data extraction |
| Data items | 10a | List and define all outcomes for which data were sought. Specify whether all results that were compatible with each outcome domain in each study were sought (e.g. for all measures, time points, analyses), and if not, the methods used to decide which results to collect. | Methods, Eligibility criteria and Data extraction; Data synthesis and statistical analysis |
| Data items | 10b | List and define all other variables for which data were sought (e.g. participant and intervention characteristics, funding sources). Describe any assumptions made about any missing or unclear information. | Methods, Data extraction; Table 1 |
| Study risk of bias assessment | 11 | Specify the methods used to assess risk of bias in the included studies, including details of the tool(s) used, how many reviewers assessed each study and whether they worked independently, and if applicable, details of automation tools used in the process. | Methods, Risk of bias assessment |
| Effect measures | 12 | Specify for each outcome the effect measure(s) (e.g. risk ratio, mean difference) used in the synthesis or presentation of results. | Methods, Data synthesis and statistical analysis |
| Synthesis methods | 13a | Describe the processes used to decide which studies were eligible for each synthesis (e.g. tabulating the study intervention characteristics and comparing against the planned groups for each synthesis (item #5)). | Methods, Data synthesis and statistical analysis; Supplementary Table 3 |
| Synthesis methods | 13b | Describe any methods required to prepare the data for presentation or synthesis, such as handling of missing summary statistics, or data conversions. | Methods, Data synthesis and statistical analysis; Supplementary Tables 4A-4B |
| Synthesis methods | 13c | Describe any methods used to tabulate or visually display results of individual studies and syntheses. | Methods, Data synthesis and statistical analysis; Figures 2-3; Supplementary Figures 1-4 |
| Synthesis methods | 13d | Describe any methods used to synthesize results and provide a rationale for the choice(s). If meta-analysis was performed, describe the model(s), method(s) to identify the presence and extent of statistical heterogeneity, and software package(s) used. | Methods, Data synthesis and statistical analysis |
| Synthesis methods | 13e | Describe any methods used to explore possible causes of heterogeneity among study results (e.g. subgroup analysis, meta-regression). | Methods, Data synthesis and statistical analysis; Supplementary Table 6 |
| Synthesis methods | 13f | Describe any sensitivity analyses conducted to assess robustness of the synthesized results. | Methods, Data synthesis and statistical analysis; Supplementary Tables 4C and 6 |
| Reporting bias assessment | 14 | Describe any methods used to assess risk of bias due to missing results in a synthesis (arising from reporting biases). | Methods, Data synthesis and statistical analysis; Certainty of evidence |
| Certainty assessment | 15 | Describe any methods used to assess certainty (or confidence) in the body of evidence for an outcome. | Methods, Certainty of evidence |
| **RESULTS** | | | |
| Study selection | 16a | Describe the results of the search and selection process, from the number of records identified in the search to the number of studies included in the review, ideally using a flow diagram. | Results, Study selection; Figure 1 (PRISMA flow diagram) |
| Study selection | 16b | Cite studies that might appear to meet the inclusion criteria, but which were excluded, and explain why they were excluded. | Results, Study selection; Supplementary Table 1 |
| Study characteristics | 17 | Cite each included study and present its characteristics. | Results, Characteristics of included studies; Table 1 |
| Risk of bias in studies | 18 | Present assessments of risk of bias for each included study. | Results, Risk of bias; Figure 2; Supplementary Tables 2 and 2B |
| Results of individual studies | 19 | For all outcomes, present, for each study: (a) summary statistics for each group (where appropriate) and (b) an effect estimate and its precision (e.g. confidence/credible interval), ideally using structured tables or plots. | Results, Results by study and Quantitative synthesis; Figure 3; Supplementary Figures 1-4 |
| Results of syntheses | 20a | For each synthesis, briefly summarise the characteristics and risk of bias among contributing studies. | Results, Quantitative synthesis |
| Results of syntheses | 20b | Present results of all statistical syntheses conducted. If meta-analysis was done, present for each the summary estimate and its precision (e.g. confidence/credible interval) and measures of statistical heterogeneity. If comparing groups, describe the direction of the effect. | Results, Quantitative synthesis; Figure 3; Supplementary Table 4C |
| Results of syntheses | 20c | Present results of all investigations of possible causes of heterogeneity among study results. | Results, Sensitivity analyses; Supplementary Table 6 |
| Results of syntheses | 20d | Present results of all sensitivity analyses conducted to assess the robustness of the synthesized results. | Results, Sensitivity analyses; Supplementary Table 4C |
| Reporting biases | 21 | Present assessments of risk of bias due to missing results (arising from reporting biases) for each synthesis assessed. | Results, Certainty of evidence; Discussion, Strengths and limitations |
| Certainty of evidence | 22 | Present assessments of certainty (or confidence) in the body of evidence for each outcome assessed. | Results, Certainty of evidence; Table 4; Supplementary Table 5 |
| **DISCUSSION** | | | |
| Discussion | 23a | Provide a general interpretation of the results in the context of other evidence. | Discussion, Summary of findings |
| Discussion | 23b | Discuss any limitations of the evidence included in the review. | Discussion, Strengths and limitations |
| Discussion | 23c | Discuss any limitations of the review processes used. | Discussion, Strengths and limitations |
| Discussion | 23d | Discuss implications of the results for practice, policy, and future research. | Discussion, Implications for clinical practice and research; Conclusion |
| **OTHER INFORMATION** | | | |
| Registration and protocol | 24a | Provide registration information for the review, including register name and registration number, or state that the review was not registered. | Title page (PROSPERO registration); Methods, Design and reporting (PROSPERO CRD420261407650) |
| Registration and protocol | 24b | Indicate where the review protocol can be accessed, or state that a protocol was not prepared. | Methods, Design and reporting (protocol registered on PROSPERO; publicly accessible via the registration record) |
| Registration and protocol | 24c | Describe and explain any amendments to information provided at registration or in the protocol. | Methods, Data synthesis and statistical analysis (post hoc REML and HKSJ analyses and inclusion of the observational cohort noted as not pre-specified) |
| Support | 25 | Describe sources of financial or non-financial support for the review, and the role of the funders or sponsors in the review. | Funding statement |
| Competing interests | 26 | Declare any competing interests of review authors. | Conflicts of interest |
| Availability of data, code and other materials | 27 | Report which of the following are publicly available and where they can be found: template data collection forms; data extracted from included studies; data used for all analyses; analytic code; any other materials used in the review. | Data availability; Supplementary Tables 4A-4C |
